# Managing the failing renal allograft: navigating a complex topography

**DOI:** 10.3389/fneph.2024.1223114

**Published:** 2025-06-05

**Authors:** Elizabeth A. Kendrick

**Affiliations:** David Geffen School of Medicine, University of California, Los Angeles, Los Angeles, CA, United States

**Keywords:** kidney transplantation, kidney transplant failure, kidney transplant complications, return to dialysis, immunosuppression withdrawal, kidney transplant outcomes, immunosuppression, kidney retransplantation

## Abstract

Recipients of kidney transplants often outlive the function of the renal allograft will need ESRD management. Patients face a higher risk of mortality in the period of transition from failing allograft to dialysis. Long term risk of cardiovascular complications and risk of infections and cancer with use of long-term immune suppression contribute to poor outcomes. Patients with failing transplants appear to have poorer control of CKD complications and are more likely to initiate hemodialysis using a catheter. Outcomes of peritoneal dialysis in the setting of the failing allograft in general are equivalent to hemodialysis. Management of these patients in transplant center clinics specifically focused on patients with failing allografts may have benefit, but maximal utility has yet to be demonstrated. Patients with failed transplants can have a survival benefit with retransplant, even in older patients. There may not be a benefit to retransplant in patients older than 70 years of age. Patients with failing renal grafts should be assessed as to whether they are potential candidates for retransplant prior to needing to start dialysis to allow for identification of a living kidney donor or to be listed as soon a possible on the kidney transplant wait list as to minimize the wait time on dialysis. Decisions regarding reduction of immunosuppression once the patient has started dialysis should be made with guidance from the transplant center in the context of patient-centric factors such as candidacy for retransplant and minimizing complications of long-term immunosuppression.

## Impact of loss of graft function and transition to ESRD care

Transplant recipients whose grafts have failed have been shown in several studies to have a higher risk of death after returning to dialysis compared to transplant-naïve patients on dialysis (1, 2). Using data from the Dialysis Outcomes and Practice Patterns Study (DOPPS), Perl et al. (3) found that patients on dialysis after a failed transplant had a relative risk of death of 1.3 compared to dialysis patients listed for a first transplant, despite patients with a failed transplant being younger and less likely to be diabetic. Additionally, using data from the DOPPS study, Gill et al. (4) reported that the majority of deaths in this group were due to cardiac (36%) and infectious (17%) causes. Despite a significant proportion of deaths being from infectious causes, non-immunologic factors—specifically cardiovascular risk factors such as diabetes, peripheral vascular disease, heart failure, and smoking—were more predictive of mortality than immunological and transplant-related factors. In some studies, the likelihood of dying on the transplant waitlist after the loss of renal transplant function was nearly as high as receiving another transplant (5, 6). However, not all reports have shown poorer survival rates after renal graft failure. In data from the national French Renal Epidemiology Information Network (REIN), Mourad et al. found similar survival in patients with failed transplants on dialysis compared to transplant-naïve patients (7). It was postulated that better outcomes compared to other studies might be due to differences in health systems and access to care; in the example of France, the context of universal health care access within a national health care system and established care models within designated nephrology units could contribute to improved patient outcomes.

Patients with failed transplants returning to dialysis appear to have fewer well-controlled complications of CKD as compared to transplant-naïve patients on dialysis, and this may contribute to the risk of poor outcomes. Lower rates of control of hyperphosphatemia and metabolic acidosis have been reported (6, 8, 9), as have increased rates of hypoalbuminemia and erythropoietin resistance, leading to inadequate correction of anemia (6, 8, 10). Erythropoietin resistance may be due to a chronic inflammatory state from the presence of the failed allograft in the context of tapered or discontinued immunosuppression. In one report, coronary flow reserve was used to assess endothelial dysfunction (ED) in peritoneal dialysis (PD) patients. ED was more prominent among patients with failed transplants on PD than transplant-naïve patients (11). Therefore, apart from the risks of long-term immunosuppression, the chronic inflammatory state maintained by the retained allograft is likely playing a role in the increased risk of mortality in this group of patients.

An impediment to achieving optimal care in the setting of a failing allograft may be in part due to the emphasis on maintaining allograft function in the setting of the transplant clinic and the relative lack of acceptance of the inevitability of eventual graft failure on the part of the patient as well as possibly the provider. As such, some transplant centers have developed dedicated clinics focused on the CKD management of transplant recipients with failing allografts. Arshad et al. (12) reported on the outcomes of a dedicated “low clearance” transplant clinic (LCTC) based in the United Kingdom (UK) for transplant recipients with an eGFR <20 ml/min, compared with patients with similar graft function who were managed in a general transplant clinic. In the setting of a LCTC, when compared to a general transplant clinic, a significantly greater proportion of patients had documented discussions of hepatitis vaccination status (63% vs. 17%), counseling regarding dialysis modality (98% vs. 55%), and documented discussion regarding re-transplantation (80% vs. 58%). However, there was no difference in mortality or control of blood pressure, anemia, or metabolic parameters between the groups. There was also no difference in the rate of transplantation between the two groups. Another report, also based in the UK, following patients with a failing allograft and an eGFR less than 30 ml/min in an LCTC, demonstrated similar better documentation of counseling and transplant workup compared to a center without such follow-up (13). There was no difference seen in patient survival, control of CKD parameters, type of dialysis modality, or use of tunneled catheters. Transplant listing rates were also equivalent. The authors note that the relatively small number of patients in the study, with a relatively short observation time, may account for the lack of apparent impact on measures such as patient mortality, and larger studies are warranted to determine if such an approach could result in better patient outcomes. It was postulated that initiating follow-up in a dedicated clinic at a higher eGFR may be more likely to impact patient outcomes. Though not the specific focus of the report, structured management of CKD patients in general and specifically those with failing renal allografts, akin to dedicated clinics discussed above, within a national health care system such as in France, may contribute to apparent better outcomes in these patients (7).

There is evidence that the rate of eGFR decline is more rapid in the failing allograft than in native kidney CKD, which can impact CKD management, dialysis planning, and access to referral. However, there is no evidence that an earlier return to dialysis based on a higher eGFR (>10 or >15 ml/min/1.73m2) in the absence of other indications for dialysis initiation improves outcomes. In one study, an earlier return to dialysis therapy in patients with failing renal grafts was correlated with worse dialysis survival, especially in the healthiest and younger patients (14, 15). Yet, earlier dialysis starts may in fact be related to the presence of factors indicating poorer overall clinical status, such as worse nutritional status, volume overload, metabolic acidosis, or a higher burden of comorbid conditions.

## Management of vascular access

Patients with failed allografts starting hemodialysis have a higher rate of central venous catheter (CVC) use as initial access compared to those with native kidney ESRD on dialysis. The rates of catheter use for initial access have been reported to range from 40% to 80% of patients (16–19). High rates of catheter usage occur even with documentation of early nephrology referrals. Haq et al. (19) reported that of 83 patients with failing transplants eventually requiring dialysis, and who had established early referral to a nephrologist, only 32% had documented discussions regarding renal replacement therapy, and 24% had vascular access referrals. Of the 34 patients without preexisting vascular access starting HD, 88% began HD with a CVC, while 11% started with an AV fistula. The low rates of permanent vascular access at the time of dialysis may be partly due to a more rapid decline in eGFR in these patients or reluctance on the part of the patient or provider to “give up” on the kidney.

Patients with failing allografts may have an AV fistula that has thrombosed and is no longer functional, complicating vascular access planning. In one study of 221 patients with failed renal allografts returning to dialysis who had a history of a functioning forearm AVF, 112 (51%) had thrombosed from 1 year to 8 years previously (20). Successful reconstruction of AVFs was accomplished in 73% of these patients, although the primary patency of reconstructed AVFs was 58% at 1 year and 44% at 2 years, somewhat lower than the rates reported in the general ESRD population (approximately 70% at 1 year and 50%–60% at 2 years).

## Choice of dialysis

The choice of dialysis modality should be based on patient preference and physician input; there is no evidence that having had a failed transplant dictates the choice of modality. There are relatively lower rates of use of peritoneal dialysis compared to transplant -naïve patients with end-stage renal disease, possibly due to concerns about modality failure from infection or prior abdominal surgery. However, several retrospective studies have shown that patient survival on PD after a failed transplant is equivalent to that on hemodialysis (21–24). Some studies noted a higher rate of technique failure in failed transplant patients, not associated with peritonitis but with adequacy and/or ultrafiltration failure (21, 22, 24). More rapid loss of residual renal graft function, impacting adequacy, may contribute to technique failure. Higher peritoneal transport was observed in patients with failed allografts on PD, contributing to technique failure (25). It was postulated that high transport status is associated with the effects of the chronic inflammatory state induced by the failed allograft on the peritoneal membrane. Conversely, another report found that peritoneal transport characteristics were unchanged compared to transport characteristics in the same patients prior to kidney transplantation (26). Moreover, Rodrigues et al. (27) found that fast transport status in transplant-naïve PD patients was not associated with inflammatory biomarkers such as CRP, IL-6, and albumin. Higher rates of peritonitis in failed transplant patients were reported in some retrospective observational studies (22, 24), but not all (21). A meta-analysis of 12 small retrospective studies concluded that patients with failed transplants did not have an increased risk of mortality, peritonitis, or technique failure in PD compared to transplant-naïve patients (28). Therefore, PD should be considered a viable dialysis modality in the setting of a failed allograft.

## Referral for re-transplantation

Patients with a failing allograft may be potential candidates for re-transplantation. Approximately 12% of the national kidney transplant waitlist in the United States consists of candidates awaiting re-transplantation. Studies have shown a survival benefit for those undergoing a second transplant compared to remaining on dialysis (29, 30), although this benefit diminishes with longer wait times for another transplant. Older patients (>65 years of age) are an increasing proportion of re-transplant candidates. These older recipients also experience a survival benefit compared to remaining on dialysis (31, 32); however, the benefit appears to be lost past age 70 (30). Reported graft and patient survival of older transplant recipients undergoing repeat kidney transplantation is similar to that of older transplant recipients after first transplant. When compared to younger transplant recipients, older patients undergoing re-transplantation are more likely to have CKD due to polycystic kidney disease or glomerulonephritis, and less likely to have diabetes and vascular disease as comorbid conditions. This difference in comorbid issues may contribute to relatively good outcomes in this setting (31).

Receiving a transplant prior to requiring dialysis [i.e., pre-emptive kidney transplant (PKT)] provides superior patient and graft survival for those undergoing re-transplantation, similar to those receiving their first transplant. However, the availability of a living kidney donor is required to achieve this dialysis likely has the greatest impact on improved outcomes. The prognosis of the renal graft in re-transplant patients may be inferior compared to patients receiving a first transplant, but reports have been mixed. Some studies have shown that graft survival in re-transplant patients is equivalent to those receiving a first transplant, while other reports (33, 34) have shown worse survival. For example, Trebern-Launay et al. (35), in an observational study using a French cohort, found that while early graft survival appeared equivalent, significantly poorer survival of the renal graft in re-transplant patients became apparent after several years. Cumulative rejection rates were equivalent for re-transplant patients and those receiving first transplants; however, rejection in the re-transplant group was more likely to be severe, and this may negatively impact long-term outcomes.

Assessment of candidacy for a repeat transplant should occur early to allow for the identification of possible living kidney donors, maximizing the chance of a pre-emptive kidney transplant (PKT) and minimizing time on dialysis for a deceased donor kidney. Since patients with a failing kidney graft have the potential for rapid loss function, it is recommended that discussion surrounding consideration for retransplant be initiated when GFR is around 30. This approach can optimize identification of potential living donors which may increase likelihood of ‘pre-emptive’ transplant prior to need for dialysis as well as listing prior to dialysis need in order to maximize ‘wait time’ on the deceased donor list. This timing helps identify living kidney donors before dialysis initiation and minimizes dialysis duration before re-transplantation. In an observational study of data from the US Renal Data System, Clark et al. (36) found that transplant-failure patients had a higher likelihood of being waitlisted compared to transplant-naïve ESRD patients. This may be due to their awareness of transplantation benefits and familiarity with the evaluation process. In addition, patients with failed transplants often have higher levels of HLA antibodies (i.e., are more “sensitized”) than those listed for their first transplant (37). The current kidney allocation system in the United States gives priority points to highly sensitized patients, which may further benefit re-transplant patients. Despite these factors that could contribute to unequal access to transplantation, Clark et al. (36) found that transplant rates were equivalent between transplant-naïve and transplant-failure patients. However, inequities in access persist; patients with reduced employment status and lower education, black patients, and men were less likely to receive PKT, similar to trends observed in first-time transplant candidates (38).

The etiology and timing of prior graft loss should be considered when evaluating patients for another transplant. If graft loss was due to the recurrence of native kidney disease, the patient might be at high risk for subsequent graft loss, especially if the recurrence occurred early in the prior transplant. This risk assessment may influence whether a living donor or a deceased donor should be considered for the next transplant. Additionally, renal graft loss due to rejection, specifically antibody-mediated rejection, is highly associated with medication non-adherence (MNA) (39). Patients with a history of immunologic graft loss should be assessed for past and current MNA to assess the risk of rejection in a subsequent transplant. Pre-transplant MNA is predictive of post-transplant non-adherence, and assessments of self-efficacy and problem-solving skills are correlated with lower MNA rates (40). Patients at higher risk of subsequent graft loss due to recurrent kidney disease or immunologic graft loss related to MNA might not be ideal candidates for living donor transplants.

## Management of immunosuppressive drugs after renal allograft failure

Patients with failed allografts who have initiated dialysis should be evaluated for the reduction of immunosuppressive drugs. Intermediate reductions may have already occurred during the course of allograft failure. Continued use of immunosuppressive drugs can potentially increase the risk of infectious disease complications or cancer post-renal allograft failure. Conversely, withdrawing immunosuppression can cause morbidity related to rejection in the non-functioning allograft (graft intolerance syndrome) or increase allosensitization in patients who may be candidates for another transplant. Therefore, specific actions regarding the timing and extent of immunosuppression reduction must take into consideration individual patient factors.

In the absence of definitive studies and agreed-upon protocols, A survey of transplant center regarding their approach of withdrawal of immunosuppression after renal graft failure showed significant variation between centers (41): at one year after dialysis initiation, in 5% of programs no patients had been taken off immunosuppression drugs, while in 40% of programs all patients had been taken off immune suppression. Overall, approximately 70% of patients were reported as being off all immunosuppression approximately 1 year after starting dialysis. Factors influencing the decision to stop or continue immunosuppression include the presence of ongoing signs or symptoms of rejection, plans for re-transplantation, history of infections, residual urine output, history of rejection, and the cost of medication. Other concerns include the risk of adrenal insufficiency from discontinuing steroids, history of cancer, risk of allosensitization, and the surgical risk associated with nephrectomy of the failed graft.

There was also variation reported in the order of weaning immunosuppression drugs after graft failure. Approximately 58% of programs reported weaning off the antimetabolite drug (mycophenolate or azathioprine) first, while 38% reported tapering off the calcineurin inhibitor (tacrolimus or cyclosporine) first. Patients on chronic prednisone were generally left on it until other drugs were weaned off.

In a more recent survey of practice patterns in US transplant centers for immunosuppression withdrawal (42), 73% of respondents stopped the antimetabolite drug first, 12% stopped the calcineurin inhibitor, and 2% stopped steroids. Thirteen percent of respondents did not have a unified protocol. The availability of a living kidney donor for re-transplantation impacted the approach toward immunosuppression drug changes: 21% of respondents continued the current immunosuppression regimen without change, 32% continued the current drugs but at a lower dosage, and 38% stopped the antimetabolite while continuing low-dose CNI and steroids. The availability of a living kidney donor was considered the most important factor in decisions regarding decreases in immunosuppression drug dosing. Other factors, in decreasing order of importance, included the risk of infection, risk of developing sensitization, frailty, medication side effects, risk of cancer, urine volume, other comorbid conditions, and age.

The lack of consistent and agreed-upon guidelines for immunosuppression withdrawal is reflected in the varied outcomes reported in studies on the management of immunosuppressive medications in the setting of failed transplants. These reports are generally based on small, retrospective studies and are often confounded by the absence of specific information regarding the weaning of particular immunosuppression drugs and the rate of weaning. Older studies showed an increased risk of infectious disease events or hospitalization with the continuation of immunosuppression beyond six months after graft failure and the initiation of dialysis (43, 44). van Leeuwen et al. (45) showed a decreased risk of cancers with confirmed infectious causes, such as Kaposi’s sarcoma, when immunosuppression was discontinued after graft failure, although this did not apply to all cancer types. The risk of other cancers, especially those related to ESRD, such as cancers of the kidney and urinary tract, remained elevated. More recent studies have generally not shown an increase in infectious disease complications or cancers in patients who continued on and weaned off immunosuppression (46–48). Some studies found an association between the continuation of immunosuppression and higher mortality on dialysis (47, 48). Conversely, Casey et al. (49) found an increase in mortality with rapid weaning of immunosuppression in less than 3 months, suggesting that this group was selected for comorbid status, leading to the decision to taper immunosuppression rapidly. Several retrospective studies have shown that prolonging the withdrawal of immunosuppression appears protective against allosensitization (49–52). Casey et al. found that 66% of patients remained non-sensitized if immunosuppression weaning occurred more than 3 months after allograft failure, compared to 30% if immunosuppression was weaned off in less than 3 months.

Elgenidy et al. (53) performed a meta-analysis of 10 retrospective cohort studies assessing the weaning of immunosuppression in patients with a failed allograft. Three studies were considered good quality according to the Newcastle-Ottawa Scale guidelines, while seven were of fair quality due to a lack of selection adjustment, comparability, or inadequacy of the follow-up period. Early withdrawal of immunosuppression was defined as discontinuation of immunosuppression in <3 months or <6 months after graft failure, depending on the study. When analyzed together, there was no significant difference in mortality, infections, decreased risk of sensitization, or need for allograft nephrectomy between patients whose immunosuppression was withdrawn within 3 or 6 months of graft failure and those who continued immunosuppression beyond this period.

Knoll et al. (54) recently reported findings from a prospective multicenter study involving 269 patients across 16 Canadian transplant centers on the outcomes of immunosuppression withdrawal after renal allograft failure. Patients were enrolled within 3 weeks of starting dialysis, and the median follow-up was 558 days. At study entry, 97% of patients were taking immunosuppression. During follow-up, 15% discontinued all immunosuppression after a median of 361 days. At 2 years of follow-up, 60% of patients were still taking prednisone, nearly 40% were taking a calcineurin inhibitor (either cyclosporine or tacrolimus), and 25% were taking an antiproliferative agent (mycophenolate or azathioprine).

The continuation of immunosuppression was not associated with a higher risk of hospitalized infection, nor was it associated with a lower risk of acute rejection of the failed allograft (i.e., graft intolerance syndrome). However, continuation of immunosuppression was associated with a lower risk of death compared to discontinuation of all immunosuppression or use of prednisone only, with an adjusted hazard ratio of 0.4. This lower risk of death may be due to the suppression of chronic inflammation by continued use of immunosuppressant drugs. Conversely, patients off immunosuppression may have had significant comorbidities leading to the discontinuation of immunosuppression and a higher risk of death. Anti-HLA antibodies increased in all patients; class I and class II panel-reactive antibody (PRA) increased to a greater extent in patients off all immunosuppression or only on low -dose prednisone within the first 12 months of the study compared to those continued on immunosuppression. However, after 24 months of observation, the differences in class I and class II PRA were not significant between the two groups. The study authors felt that their findings implied that even though patients were continued on immunosuppression, the dosing of the drugs was insufficient to prevent sensitization. They proposed that higher dosing of immunosuppression drugs in the context of graft failure may be needed to prevent sensitization in patients who may be candidates for another transplant.

Taken together, reports of outcomes from immunosuppression withdrawal suggest that if a patient is not a candidate for re-transplantation, immunosuppression can be safely tapered and discontinued within 6 months after initiating dialysis. There does not appear to be any benefit to continuing immunosuppression beyond this period, nor is there any advantage to a more rapid taper in terms of reducing cancer or infection risk. In fact, a rapid taper of immunosuppression may increase the risk of graft intolerance syndrome.

For patients who are candidates for re-transplantation, the risk of sensitization may be reduced by continuing immunosuppression at higher doses for a longer period. If a patient with a failed graft has a potential living kidney donor or anticipates a short wait for a deceased donor organ, maintaining minimal reductions in chronic immunosuppression may be considered.

Graft intolerance syndrome in the context of immunosuppression withdrawal can cause significant morbidity, often necessitating increased immunosuppression—typically high-dose steroids—on a temporary basis. This situation may also require an allograft nephrectomy, which carries additional morbidity and mortality risks. Avoiding graft intolerance syndrome is a key goal in managing immunosuppression reduction. However, clear guidelines on the recommended rate of immunosuppression reduction are lacking. Reported experience suggests no apparent benefit to prolonging withdrawal beyond 3 to 6 months.

Some subgroups of patients may be at higher risk for graft intolerance syndrome upon withdrawal of immunosuppression and should be considered for early referral for graft nephrectomy before attempting major reductions in immunosuppression. Patients experiencing early graft failure within the first few years after transplantation due to rejection or other acute causes are likely to require nephrectomy, as they are at high risk for graft intolerance syndrome upon immunosuppression withdrawal. Conversely, patients with slow progression of CKD over months to years prior to graft failure are often able to discontinue immunosuppression with a lower risk of developing graft intolerance and a possible need for nephrectomy. The role of allograft nephrectomy in the context of the failed allograft has been more extensively reviewed in previous editions of this journal (55).

Formal guidelines for the management of patients with a failed allograft, including the management of immunosuppression and CKD complications, have been provided by the British Transplantation Society (56) and, more recently, the American Society of Transplantation (AST) (57). Since a major consideration regarding weaning immunosuppression is whether the patient is a candidate for re-transplantation and minimizing the risk of further sensitization, recommendations suggest tapering immunosuppression more slowly in those patients compared to those who are not considered candidates for another transplant. Specific recommendations for the rate of reducing immunosuppression in re-transplant candidates are more conservative. Nevertheless, by 12 months, both groups of patients are on low amounts of immunosuppression, with consideration for total discontinuation at 12 months even in re-transplant candidates. Maintaining a higher dose of immunosuppression may be indicated for re-transplant candidates with living kidney donors to ensure adequate immunosuppression and minimize allosensitization. **Table 1** summarizes general recommendations for reducing immunosuppression as put forth by the AST. Patients should be monitored by the transplant center throughout the immunosuppression tapering period to make decisions on changes in drug dosing and to monitor for complications.

**Table 1:** Recommendations for tapering of immunosuppression.

	Candidate for re-transplant	Not a candidate for re-transplant
**Initial**	Reduction in anti-metabolite by 50%, maintain CNI ± low dose prednisone	Stop antimetabolite
**3 months post DI**	Stop anti-metabolite, maintain low dose CNI ± low dose prednisone	Taper CNI by 50%
**6 months post DI**	Reduce CNI by 50% ± low dose prednisone	Maintain on low dose CNI and/or low dose prednisone therapy for 6-12 months in coordination with transplant nephrology
**9 months post DI**	Consider additional reduction in CNI or maintenance of prednisone 5 mg	Monitor for graft intolerance syndrome
**12 months post DI**	Consider cessation of all immunosuppression if not signs of graft intolerance syndrome and no significant increase in cPRA value	Monitor patient every 3-6 months until patient is off immunosuppression
**Beyond 12 months**	Continue to monitor for sensitization while wait-listed and for signs of toxicity from immunosuppression	

DI, dialysis initiation; CNI, calcineurin inhibitor.

Adapted from Lubetzky et al, American Journal of Transplantation, 2021, 21:2937-2949.

## Coordination of care

Transplant recipients with failing grafts may find themselves caught between two worlds: that of the transplant center and that of community nephrology, which oversee their CKD care and transition to dialysis. As discussed, these patients face issues requiring complex management that very likely benefit from close coordination between their providers. In particular, decisions regarding the tapering of immunosuppression once the patient has started dialysis would benefit from regular input from the transplant program. However, in reality, coordination of care may not be optimized. In a survey conducted by a workgroup of the American Society of Transplantation (AST), follow-up of patients with failed allografts returning to dialysis varied considerably between transplant centers: 36% of respondents did not have a unified follow-up protocol, 21% saw patients every 3 months to 6 months until they were off immunosuppression, 17% saw patients only once after starting dialysis, and 11% did not see patients once they had started dialysis (42). Therefore, recommendations for optimal care of patients with failing transplants include ongoing coordination of care between the transplant center and the local nephrology provider of the patient, even after the patient has started dialysis. As previously discussed, small-scale studies on the outcomes of dedicated “low clearance” transplant clinics for patients with failing allografts did not show improved control of CKD complications or re-transplant rates. However, there may be an opportunity for patient benefit in terms of assessment for re-transplant, reducing morbidity, and optimizing the transition to ESRD care with wider use of this approach initiated earlier at an earlier stage of CKD. **Table 2** summarizes the AST working group recommendations for the follow-up of patients with failing grafts.

**Table 2:** Management of the patient with a failing allograft.

	Candidate for re-transplant	Not a candidate for retransplant
Stable transplant function, eGFR >20 ml/min/m^2^	Close monitoring of levels of immunosuppression and side effects Optimize CKD management including BP control, anemia, proteinuria, secondary hyperparathyroidism	Establish joint management approach with general nephrologist Continue close monitoring at transplant center Close monitoring of levels of immunosuppression and side effects Optimize CKD management including BP control, anemia, proteinuria, secondary hyperparathyroidism
Failing transplant with declining function	Refer for re-listing when eGFR approached 20 Establish baseline PRA value Living Donor Champion Optimize wait-list management Discuss options for decreasing time to transplantation Referral for vascular access if there is no living donor Referral to general nephrology for preparation for dialysis Consider reduction in immunosuppression to decrease side effects and complications Maintain CNI trough in the low therapeutic range	Establish vascular access Continue transition of care to general nephrology Coordinate reduction of immunosuppression over time Reduction in anti-metabolite by 50% Maintain CNI ± low dose prednisone Monitor for graft intolerance syndrome
Failed allograft with return to dialysis	Primary management with general nephrology Monitor cPRA every 3-6 months Taper of immunosuppression	Primary management with general nephrology Taper of immunosuppression

Adapted from Lubetzky et al, American Journal of Transplantation, 2021, 21:2937-2949.

In summary, patients with failing renal allografts face a period of potential clinical instability with an increased risk of death once they start dialysis. Morbidity can increase due to the cumulative effect of cardiovascular risk factors and complications, as well as an elevated risk of cancer and infection in the context of chronic immune suppression. Early assessment of re-transplant candidacy can minimize time on dialysis if a living donor is available or if the patient can be listed prior to starting dialysis. Early referrals for dialysis access can help avoid the initiation of hemodialysis using a central catheter. Patients with failed transplants should be considered for peritoneal dialysis.

The management of reduction and eventual discontinuation of immunosuppression should be done in consideration of whether the patient is a candidate for re-transplant and should be directed by the transplant center with continued follow-up of the patient. Whether patients can benefit from a dedicated “low clearance” clinic managing issues related to the failing transplant deserves further study.

## Author contributions

The author confirms being the sole contributor to this work and has approved it for publication.
